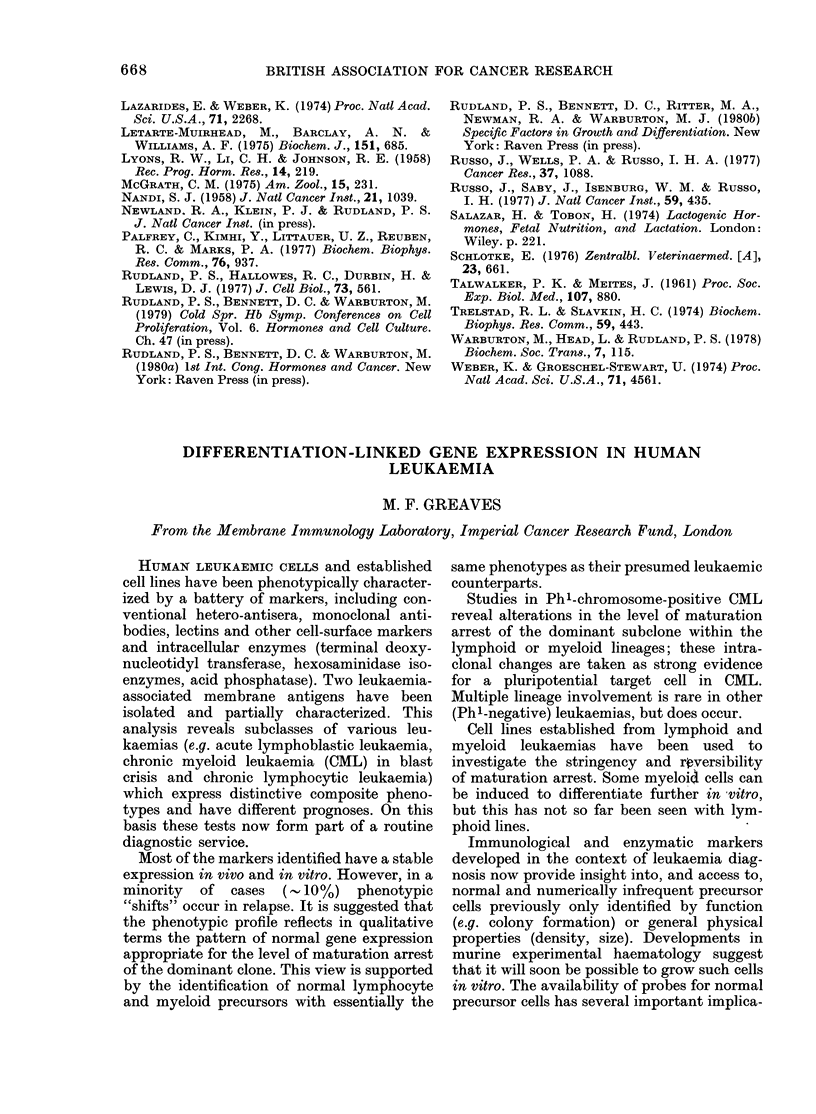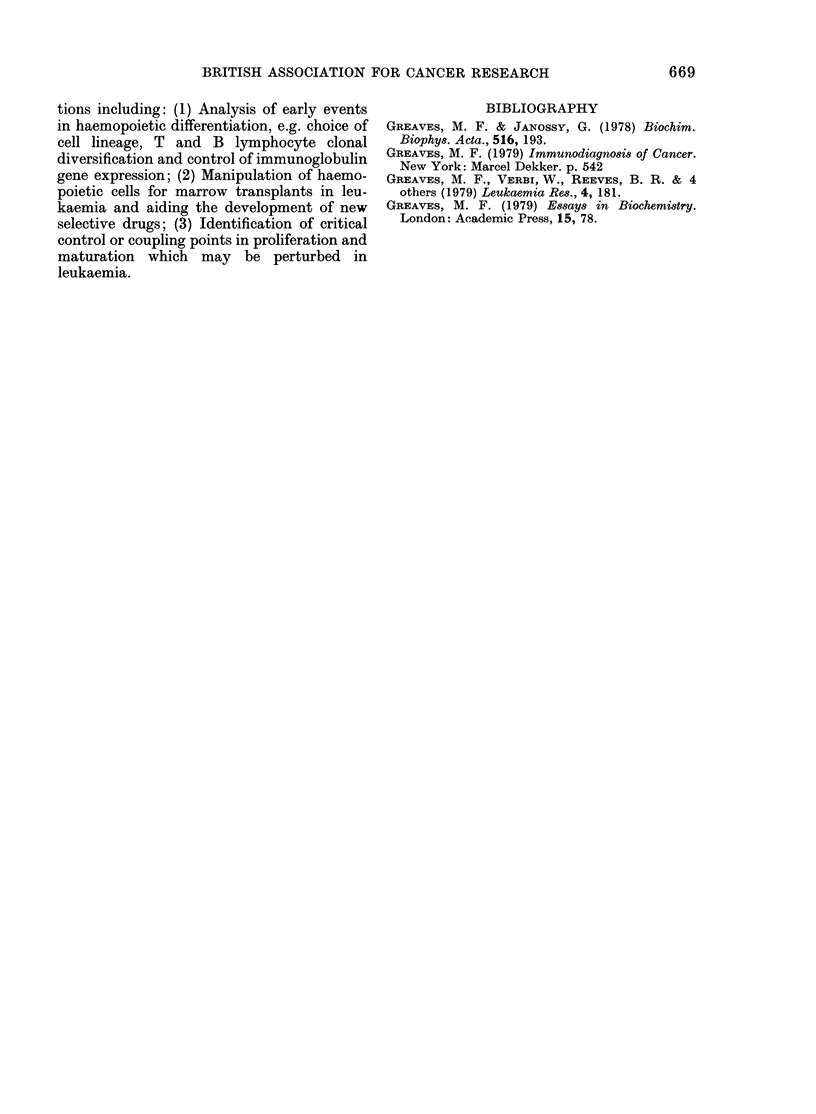# Differentiation-Linked Gene Expression in Human Leukaemia

**Published:** 1980-04

**Authors:** M. F. Greaves


### DIFFERENTIATION-LINKED GENE EXPRESSION IN HUMAN LEUKAEMIA

M. F. GREAVES

*From the Membrane Immunology Laboratory, Imperial Cancer Research Fund, London*

HUMAN LEUKAEMIC CELLS and established cell lines have been phenotypically characterized by a battery of markers, including conventional hetero-antisera, monoclonal antibodies, lectins and other cell-surface markers and intracellular enzymes (terminal deoxynucleotidyl transferase, hexosaminidase isoenzymes, acid phosphatase). Two leukaemia-associated membrane antigens have been isolated and partially characterized. This analysis reveals subclasses of various leukaemias (*e.g.* acute lymphoblastic leukaemia, chronic myeloid leukaemia (CML) in blast crisis and chronic lymphocytic leukaemia) which express distinctive composite phenotypes and have different prognoses. On this basis these tests now form part of a routine diagnostic service.

Most of the markers identified have a stable expression *in vivo* and *in vitro*. However, in a minority of cases ($\sim$10%) phenotypic "shifts" occur in relapse. It is suggested that the phenotypic profile reflects in qualitative terms the pattern of normal gene expression appropriate for the level of maturation arrest of the dominant clone. This view is supported by the identification of normal lymphocyte and myeloid precursors with essentially the same phenotypes as their presumed leukaemic counterparts.

Studies in Ph$^1$-chromosome-positive CML reveal alterations in the level of maturation arrest of the dominant subclone within the lymphoid or myeloid lineages; these intraclonal changes are taken as strong evidence for a pluripotential target cell in CML. Multiple lineage involvement is rare in other (Ph$^1$-negative) leukaemias, but does occur.

Cell lines established from lymphoid and myeloid leukaemias have been used to investigate the stringency and reversibility of maturation arrest. Some myeloid cells can be induced to differentiate further *in vitro*, but this has not so far been seen with lymphoid lines.

Immunological and enzymatic markers developed in the context of leukaemia diagnosis now provide insight into, and access to, normal and numerically infrequent precursor cells previously only identified by function (*e.g.* colony formation) or general physical properties (density, size). Developments in murine experimental haematology suggest that it will soon be possible to grow such cells *in vitro*. The availability of probes for normal precursor cells has several important implications including: (1) Analysis of early events in haemopoietic differentiation, e.g. choice of cell lineage, T and B lymphocyte clonal diversification and control of immunoglobulin gene expression; (2) Manipulation of haemopoietic cells for marrow transplants in leukaemia and aiding the development of new selective drugs; (3) Identification of critical control or coupling points in proliferation and maturation which may be perturbed in leukaemia.

### BIBLIOGRAPHY

GREAVES, M. F. & JANOSSY, G. (1978) *Biochim. Biophys. Acta.*, **516**, 193.

GREAVES, M. F. (1979) *Immunodiagnosis of Cancer.* New York: Marcel Dekker. p. 542

GREAVES, M. F., VERBI, W., REEVES, B. R. & 4 others (1979) *Leukaemia Res.*, **4**, 181.

GREAVES, M. F. (1979) *Essays in Biochemistry.* London: Academic Press, **15**, 78.